# A pandemia reveladora: limites e reinvenções da epidemiologia

**DOI:** 10.1590/0102-311XPT102626

**Published:** 2026-07-27

**Authors:** Moisés Goldbaum, Euclides Ayres de Castilho

**Affiliations:** 1 Faculdade de Medicina, Universidade de São Paulo, São Paulo, Brasil.

Antecipando a resenha que fazemos do livro *Epidemiologia no Pós-pandemia: De Ciência Tímida a Ciência Emergente* de Naomar de Almeida Filho ^1^, algumas considerações merecem ser feitas. De um lado, a notabilidade, a capacidade e a integridade científicas do autor, cujo reconhecimento está absolutamente inscrito na comunidade técnico-científica do setor saúde. De outro, a outorga do Prêmio Jabuti 2025 reforça a excelência da qualidade do trabalho, o que constitui característica marcante dos produtos intelectuais por ele oferecidos.

Na abertura do livro, as ponderações registradas nos prefácios e na apresentação da primeira edição, feitas respectivamente por José Ricardo Ayres, Mario Testa e Juan Samaja, já *de per si* assinalam os instigantes diálogos propostos pelo autor nos capítulos.

Longe de ser um livro didático e formal sobre epidemiologia, trata-se de formulações desafiadoras, inovadoras e provocativas sobre a epidemiologia, como ciência. O autor elabora seu pensamento com vistas a evidenciar a superação da aparente timidez da epidemiologia e a revelar seu potencial como ciência densa e exuberante. Sua redação, ao registrar, de modo original, como um ensaio-depoimento-memória, dá origem a um texto cuja leitura, por vezes, assemelha-se a um colóquio travado diretamente com o leitor, por conta de seu estilo, sempre presente, de transmissão de conhecimentos científicos para usar um termo usual da moderna comunicação: amigável.

Percorrer os 23 capítulos do livro permite visualizar a trajetória da vida acadêmica de Naomar que, em muitos deles, se detém, de modo apropriado e por vezes explorando sua invejável capacidade literária, a descrever os estímulos desencadeantes das reflexões, bem como identificar personalidades científicas com as quais manteve contato. Seu olhar curioso transparece nesses contatos e estímulos dando a entender e compreender suas postulações merecedoras de atenção da comunidade epidemiológica e dos campos disciplinares da Saúde Coletiva. Seu cuidado ao descrever os capítulos é competentemente embasado na bibliografia existente, oferecendo uma extensa e generosa lista de referências bibliográficas que, por si só, se constitui em valioso acervo, facilitando o acesso aos produtos técnico-científicos nacionais e internacionais.

A leitura dos capítulos revela a preocupação de Naomar em desconstruir/reconstruir o objeto, os conceitos, os métodos, as práticas da epidemiologia e, para tanto, em vários dos capítulos, traz um amplo debate sobre a concepção de saúde e seus conceitos. Faz-se valer de um procedimento pouco usual na comunidade epidemiológica que é a interlocução com áreas diversas da ciência, como, entre outras, a filosofia/epistemologia e, particularmente, com a quase ausente atenção com as questões teóricas. A partir dessa iniciativa bem elaborada, ainda que se possa a ela se contrapor em pontos específicos (aqui vale a menção ao diálogo a que ele se propõe), ressalta-se que, como indica sua postulação, apesar de trabalhar com saúde, a epidemiologia é refém das concepções de doença, objeto primordial e elaborada baseando-se na clínica.

Ao lado de rever, analisar, criticar, inovar sobre o conceito de saúde, acompanhar os escritos expostos no livro permite identificar a preocupação do autor em debater sobre os conceitos estruturantes da metodologia epidemiológica. Assim, dedica-se a explorar categorias como objeto, risco, causação, causalidade, multi, inter e transdisciplinaridade, a epidemiologia e a clínica, entre outros pontos importantes.

Abordar o conceito saúde tal como trabalhado no campo da epidemiologia é impositivo, e no livro Naomar analisa seus aspectos de modo primordial, apontando a ausência de atenção a questões filosóficas e teóricas, bem como a insistência dos epidemiologistas em buscar a estrita quantificação de eventos. Assim, se desdobra em vários momentos a apontar caminhos pertinentes para promover reformulação e revisão dos postulados teóricos. Apoiado em cientistas de diversos campos disciplinares, dos quais nos fornece as respectivas referências, não deixa de aprofundar-se na análise das diferentes concepções utilizadas na literatura, bem como na atuação dos formuladores de política, nos epidemiologistas voltados para a pesquisa e nas formas de atuação dos profissionais de saúde. Um excerto de sua escrita reflete a preocupação do autor e orienta o leitor da necessidade de atenção: “*Em suma, se conceituamos os fenômenos de saúde-doença como processos sociais e aceitamos o pressuposto de que os processos sociais são históricos, complexos, fragmentados, orgânicos, corporais, conflituosos, dependentes e incertos, precisamos gerar os dispositivos interpretativos mais adequados para referenciar, com o devido rigor, os objetos da pesquisa científica em saúde. Para tanto, urge conceber e utilizar uma abordagem capaz de fazer jus à natureza complexa e múltipla dos processos concretos relacionados à vida, à angústia, ao sofrimento, à dor, à doença, ao cuidado, à cura e à morte - à saúde, enfim - que ocorrem em conglomerados humanos históricos*” (p. 453).

Aponta, no capítulo específico intitulado *A Questão Conceitual da Saúde*, que esta não é, enquanto objeto plural, mutante, relativo e não ontológico, questão original ou recente na ciência, apontada que já fora em 1882, por Friedrich Nietzsche. Suas indagações sobre saúde, como referido em diversos momentos do livro, lhe permitem propor encaminhamentos e desafios consistentemente elaborados para os quais se impõe a reflexão da comunidade epidemiológica e, reiterando, das disciplinas da Saúde Coletiva, bem como do setor saúde em geral.

Detém-se a analisar e rever com propriedade o conceito de risco, elemento fundamental no campo da epidemiologia. Retomando sua crítica inicial, insiste sobre a pouca atenção da perspectiva teórico-metodológica e aponta seu uso como entidade exclusivamente operacional, restringindo-se à dimensão da probabilidade, não dando conta, portanto, da complexidade que cerca os objetos da disciplina e promovendo, nas palavras do autor, “*uma redução dos sistemas, dispositivos, forças, vetores, agentes e atores que compõem estruturas complexas a elementos simples e conexões lineares*” (p. 247). Em certo momento, fundamenta suas observações contando com o seu domínio das artes culturais (outra qualidade de Naomar), fazendo-se valer da obra de Saramago (*Todos os Nomes*) e da tetralogia *Matrix*, evidenciando e reiterando a complexidade marcante da vida humana e do mundo, plena de imprecisões e de ambiguidades, cuja integração disciplinar de conhecimentos proporciona percepções mais confiáveis e convicções mais defensáveis.

Na sequência de seu pensamento, articulado às questões que postula sobre o conceito de risco, traz à baila uma ampla discussão sobre o causalismo epidemiológico. O termo causa, que tradicionalmente é entendido como associações probabilísticas de risco, se transforma, tal como se identifica no texto, num processo de naturalização, suprimindo seu caráter histórico, mostrando uma vez mais a ausência da complexidade que cerca o objeto da epidemiologia. Este tema também se vê representado quando brinda os leitores fundamentado na sua experiência e na farta citação de autores fundamentais, com uma análise das relações entre a clínica médica e a epidemiologia (curiosamente, afirma que ao entrar em contato com os protagonistas desta linha de atuação foi capturado pela doença infantil da resistência à epidemiologia, que hoje renega) na perspectiva das epidemiologias sociais.

Fazendo uso de sua rica experiência em várias posições acadêmicas e de sua dedicação a fundamentar seu inquietante e atento interesse científico, introduz no debate um assunto que não só diz respeito à epidemiologia, mas inclui o campo da Saúde Coletiva como um todo e o próprio setor saúde. Trata-se da transdisciplinaridade. Visita os termos multi, pluri, meta, inter e transdisciplinar e, em um de seus “arremates”, faz uma citação de Nicolescu: “*a transdisciplinaridade, como prefixo ‘trans’ indica diz respeito àquilo que estão ao mesmo tempo entre as disciplinas, através das diferentes disciplinas e além de qualquer disciplina. Seu objetivo é a compreensão do mundo presente, para o qual um dos imperativos é a unidade do conhecimento*” (p. 514).

A profícua produção se completa com a parte mais original do livro quando adentra a expor a teoria da holopatogênese. Discorre sobre e indica as bases de sua aplicação para o entendimento de objetos complexos e singulares, exemplificando com a pandemia/sindemia da COVID-19 para demarcar o necessário esforço teórico.

Promover a resenha de um livro com 23 capítulos e quase 700 páginas contendo rica e bem fundamentada exposição sobre a história da epidemiologia, passando pela abordagem dos conceitos que estruturam essa disciplina científica não se esgota no espaço dedicado pelos periódicos. Entretanto, ao redigi-la, constatamos que esta apreciação se configura mais como um estimulante para que este livro seja reconhecido como um importante material para orientar o debate que é impositivo nos programas de formação de recursos humanos para a saúde, de desenvolvimento científico, de prestação de serviços. Debruçar sobre os termos que estruturam a disciplina, como saúde, processo saúde-doença, causa e risco, probabilidades, transdisciplinaridade, desigualdade social em saúde, epidemiologia e clínica, epidemiologia social (que aponta com propriedade a redundância do social, haja vista o qualificativo social estar contido no seu nome - *demos*) e holopatogênese permite encontrar nele as bases estruturantes para fazer avançar, ainda mais, o diálogo/debate/crítica necessário para confirmar a importância da ciência epidemiológica que a pandemia reforçou.

## References

[1] Almeida N (2024). Epidemiologia no pós-pandemia: de ciência tímida a ciência emergente..

